# The Next Frontier in Sarcoma Care: Digital Health, AI, and the Quest for Precision Medicine

**DOI:** 10.3390/jpm13111530

**Published:** 2023-10-25

**Authors:** Bruno Fuchs, Gabriela Studer, Beata Bode-Lesniewska, Philip Heesen, on behalf of the Swiss Sarcoma Network

**Affiliations:** 1 Sarcoma Service, University Teaching Hospital LUKS, University of Lucerne, 6000 Lucerne, Switzerland; 2 Sarcoma Service, Kantonsspital Winterthur, 8400 Winterthur, Switzerland; 3 Patho Enge, SSN Reference Sarcoma Pathology, University of Zurich, 8000 Zurich, Switzerland; 4 University Hospital USZ, University of Zurich, 8000 Zurich, Switzerland

**Keywords:** digital health, artificial intelligence, value-based healthcare, sarcoma, precision medicine, benchmarking, interoperable platforms, quality indicators

## Abstract

The landscape of sarcoma care is on the cusp of a transformative era, spurred by the convergence of digital health and artificial intelligence (AI). This perspectives article explores the multifaceted opportunities and challenges in leveraging these technologies for value-based, precision sarcoma care. We delineate the current state-of-the-art methodologies and technologies in sarcoma care and outline their practical implications for healthcare providers, administrators, and policymakers. The article also addresses the limitations of AI and digital health platforms, emphasizing the need for high-quality data and ethical considerations. We delineate the promise held by the synergy of digital health platforms and AI algorithms in enhancing data-driven decision-making, outcome analytics, and personalized treatment planning. The concept of a sarcoma digital twin serves as an illustrative paradigm for this integration, offering a comprehensive, patient-centric view of the healthcare journey. The paper concludes with proposals for future research aimed at advancing the field, including the need for randomized controlled trials or target trial emulations and studies focusing on ethical and economic aspects. While the road to this transformative care is laden with ethical, regulatory, and practical challenges, we believe that the potential benefits far outweigh the obstacles. We conclude with a call to action for multidisciplinary collaboration and systemic adoption of these technologies, underscoring the urgency to act now for the future betterment of sarcoma care and healthcare at large.

## 1. Introduction

Sarcoma, a rare and heterogenous group of malignant tumors originating from mesenchymal tissues, poses unique challenges for healthcare providers and patients alike. With over 100 subtypes and often complex clinical presentations, treating sarcoma requires a multidisciplinary, data-driven approach—an approach that modern healthcare is progressively leaning towards but has not yet fully realized [1,2,3,4,5]. In terms of the state of the art, recent advancements in genomics, targeted therapies, and immunotherapy have begun to reshape the landscape of sarcoma treatment. However, these advancements are often isolated in their impact, lacking a cohesive, data-driven strategy for implementation across healthcare systems. The integration of artificial intelligence (AI) and digital health platforms represents the next frontier in this context. These technologies have the potential to synthesize large and complex datasets, from genomic information to real-world-time patient outcomes, thereby enabling more precise and personalized care. This is particularly crucial for sarcoma, given its heterogeneity and the consequent need for highly individualized treatment plans. The dawn of precision medicine has ushered in an era where treatment is personalized, not just to the disease but to the individual [6]. Yet, while the promise of precision medicine is substantial, its full realization is intricately tied to the evolution of healthcare systems towards value-based models, especially for complex conditions like sarcoma [7,8].

The notion of value-based healthcare (VBHC) emphasizes patient-centricity, focusing on metrics that matter most to the patient’s well-being. This patient-centricity must be supported by robust, real-world-time data analytics that not only gauge the quality of care but also its cost-effectiveness [9,10]. Recent advancements in digital health technologies and AI have demonstrated unprecedented potential to empower this transition, offering an innovative toolkit for data collection, management, and predictive analytics [11,12,13].

**Figure 1:**
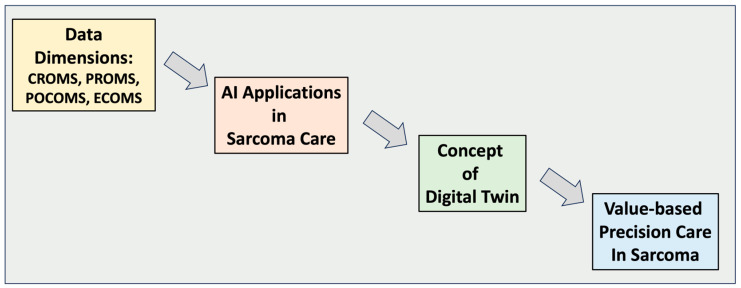
The figure depicts the evolution of sarcoma care, emphasizing patient-centricity and a data-driven approach. Digital health technologies, like Sarconector, form the foundation, streamlining patient data integration. Building on this, AI employs advanced algorithms to precisely characterize sarcoma subtypes and predict treatment outcomes. The pinnacle is the ‘digital twin’, a virtual patient profile that harnesses AI for predictive modeling and treatment optimization. CROMS = clinician-reported outcome measures; PROMS = patient-reported outcomes measures; POCOMS = patient-omics-centric outcome measures; ECOMS = economic measures.

However, the intersection of digital health and AI remains an underexplored terrain, especially in the context of sarcoma care [14]. This article aims to go beyond a mere review of existing technologies and methodologies. Instead, it seeks to offer a forward-looking perspective on how the confluence of these technologies could redefine the very essence of sarcoma care, contributing to a future where diagnosis is precise, treatment is personalized, and outcomes are continually optimized [15] (Figure 1).

In doing so, this article embarks on a visionary journey to explore the untapped potential of digital health and AI. It aims to serve as a catalyst for multidisciplinary dialogue and research, encouraging healthcare professionals, policymakers, and technologists to collaborate in transforming the future of sarcoma care—making it more precise, sustainable, and, above all, value-based.

## 2. The Vision for Value-Based Precision Care in Sarcoma

The pursuit of value-based healthcare is not merely a trend but a paradigm shift—one that brings the patient to the center of the healthcare universe [16]. In the context of sarcoma, a complex and rare malignancy, this transformation is not just aspirational but essential [15]. The heterogeneity of sarcoma, spanning multiple subtypes and clinical complexities, demands an individualized, outcomes-focused approach [17,18]. Traditional healthcare systems, largely built on a volume-based model, often fall short in providing the comprehensive, personalized care that sarcoma patients require. Value-based care in sarcoma envisions a healthcare ecosystem where every stakeholder, from surgeons and oncologists to data scientists and policymakers, collaborates to enhance the patient experience—from diagnosis to treatment and follow-up. It is an approach that goes beyond the immediate clinical outcomes to consider the patient’s quality of life, long-term well-being, and the economic sustainability of the care provided. In this envisioned ecosystem, treatment protocols are not rigid pathways but dynamic algorithms, constantly updated with real-world-time data and adapted to each patient’s unique medical history, genomic profile, and even psychosocial needs [19,20,21].

This vision is not utopian; it is attainable. Emerging technologies in digital health, coupled with advances in artificial intelligence and machine learning, offer the tools needed to actualize this vision. Imagine a future where an interoperable digital platform integrates multi-dimensional data, from medical imaging to genomic sequencing and patient-reported outcomes. These data are then processed by sophisticated AI algorithms to provide actionable insights, ranging from predicting treatment responses to estimating healthcare costs [19,22]. Moreover, the continuous benchmarking against quality indicators ensures that the care provided is not just effective but continually optimized [22].

However, the transition from vision to reality entails overcoming significant barriers—technological, ethical, and institutional. The subsequent sections of this article delve into these challenges, offering a multi-faceted perspective on how the confluence of digital health and AI can serve as the linchpin in materializing the vision for value-based care in sarcoma.

## 3. Potential of Digital Health

Digital health stands as a cornerstone in the realization of value-based care, especially in the intricate landscape of sarcoma [23,24]. The advent of technologies such as interoperable Electronic Health Records (EHRs), telemedicine, and real-world-time data platforms has enabled healthcare systems to move beyond the siloed structures of the past [19,22]. These technologies permit the seamless integration of multi-dimensional patient data—from diagnostic imaging and laboratory results to patient-reported outcomes and follow-up care metrics [25]. The role of digital health in sarcoma care is not merely auxiliary; it is transformative. For instance, telemedicine has proven to be invaluable in providing specialized sarcoma care to patients in remote locations, breaking down geographical barriers to quality healthcare. This is particularly crucial for a rare and complex disease like sarcoma, where specialized expertise may not be readily available in all regions. Interoperable EHRs, on the other hand, facilitate multi-disciplinary collaboration by allowing seamless data sharing between oncologists, radiologists, pathologists, and even primary care physicians. This is vital in sarcoma care, which often requires a multi-disciplinary approach for optimal outcomes. The EHRs can also be integrated with AI algorithms to flag potential issues or suggest alternative treatment pathways based on historical data and predictive analytics. Moreover, real-world-time data platforms can serve as a tool for continuous quality improvement. By tracking key performance indicators in real-time, healthcare providers can identify areas for improvement almost instantaneously, allowing for rapid intervention and adaptation of care protocols [19].

But the potential of digital health is not just in data collection; it is in data utilization. Advanced digital platforms can streamline the diagnostic journey, enhance treatment personalization, and even predict clinical outcomes, thereby offering a more holistic, patient-centric model of care. For example, digital platforms can automate the pre-diagnostic phase by gathering and analyzing patient history, symptoms, and preliminary test results, thereby aiding clinicians in making more accurate initial assessments. These platforms can also integrate with wearable devices that monitor patient vitals and other health metrics, providing a continuous stream of data that can be invaluable for ongoing care and monitoring. Furthermore, digital health technologies can facilitate patient engagement by providing platforms for virtual consultations, remote monitoring, and even digital therapeutics. These technologies empower patients to take an active role in their healthcare journey, thereby aligning with the principles of value-based care.

In essence, digital health technologies serve as the scaffolding upon which value-based care in sarcoma can be constructed, offering the dual advantages of operational efficiency and clinical efficacy.

## 4. Future Applications of AI in Sarcoma Care

As we look toward the horizon of sarcoma care, artificial intelligence (AI) emerges as a highly promising tool for advancing the field [26,27]. While current applications have been instrumental in diagnosis and treatment planning, the future holds even greater promise. Advanced machine learning algorithms are poised to delve into multi-omics data, offering unprecedented levels of precision in characterizing sarcoma subtypes and predicting treatment responses. The application of machine learning in sarcoma research extends beyond clinical data and can incorporate environmental, genetic, and lifestyle factors. By creating more comprehensive models that consider these variables, AI has the potential to identify new risk factors and even suggest preventative measures for at-risk populations. Deep learning techniques, a subset of machine learning, could be particularly impactful in image analysis. These algorithms can analyze complex patterns in radiological images that may be too subtle for the human eye, thereby aiding in early diagnosis and more accurate staging of the disease. This is crucial for sarcoma, where early diagnosis can significantly improve prognosis.

These algorithms could also integrate radiomic features with pathological and clinical data, refining prognostic accuracy [28]. Moreover, AI has the potential to support real-time decision making during surgeries through augmented reality interfaces, allowing for more precise surgical interventions. The introduction of natural language processing (NLP) can further enhance patient engagement by automating the analysis of patient-reported outcomes, thereby incorporating the patient’s voice directly into the care continuum. NLP’s real strength lies in its ability to convert unstructured data, such as patient narratives or free-text clinical notes, into structured data that can be easily analyzed. This is particularly valuable in sarcoma care, where patient experiences and symptoms can be highly variable and complex. By applying NLP algorithms to these unstructured data sources, healthcare providers can gain insights into patient well-being, treatment side effects, and even early indicators of complications that may not be readily apparent through traditional structured data. These structured data can then be integrated into machine learning models to improve predictive accuracy, thereby contributing to more personalized and effective treatment plans.

In a value-based healthcare framework, AI can enable more personalized, efficient, and outcome-oriented care, serving as a catalyst for transforming the ideal of precision sarcoma care into a tangible reality.

## 5. The Concept of Sarcoma Digital Twin

The notion of a “Digital Twin” in sarcoma care is a groundbreaking concept that aligns closely with the goals of precision medicine and value-based healthcare [29]. Drawing inspiration from the Swiss Sarcoma Network’s robust digital platform, the Sarconector, a sarcoma digital twin serves as a virtual replica of an individual patient’s medical profile, integrating real-world-time data including Clinical-Reported Outcome Measures (CROMS), Patient-Reported Outcome Measures (PROMS), POCOMS (patient-omics-centric outcome measures), ECOMS (economic measures), and other metrics from multiple sources like Electronic Health Records (EHR), surveys, and interviews [22,30].

The concept of a digital twin goes beyond merely storing or aggregating data; it serves as a dynamic, interactive model that evolves in real-world-time. As new clinical data become available, whether they are from imaging studies, laboratory tests, or patient-reported symptoms, the digital twin updates accordingly. This dynamic nature allows for a more nuanced understanding of the patient’s condition, thereby facilitating more informed clinical decisions. Moreover, the digital twin concept is not limited to the individual patient level. When aggregated across a population of sarcoma patients, these digital twins can serve as a rich data repository for observational studies, clinical trials, and even epidemiological research. This collective data pool can be invaluable for identifying patterns or trends in sarcoma treatment and outcomes, thereby contributing to evidence-based medicine.

By leveraging AI-driven analytical tools, the digital twin can assist in predictive modeling, optimizing treatment plans, and even simulating potential outcomes of various therapeutic strategies. This creates an innovative ecosystem for quality-centric, value-based sarcoma care, enabling iterative improvement based on ongoing assessments and benchmarking. The utility of AI in this context is multifold. For instance, machine learning algorithms can analyze the digital twin data to predict patient responses to different treatment modalities, thereby aiding in personalized treatment planning. Furthermore, natural language processing (NLP) algorithms can sift through clinical notes and patient interviews to extract valuable insights that may not be readily apparent through quantitative data alone. These AI-driven analyses can be integrated into the digital twin, providing a comprehensive, 360-degree view of the patient’s health status and treatment options. The concept of a sarcoma digital twin also has implications for healthcare economics. By providing a more accurate and personalized treatment plan, it has the potential to reduce unnecessary tests and treatments, thereby contributing to cost-effectiveness and sustainability in healthcare systems. In doing so, the concept of a sarcoma digital twin pushes the frontier of what is possible in delivering personalized, effective, and efficient care to sarcoma patients.

## 6. Roadmap to the Future

The Swiss Sarcoma Network’s comprehensive roadmap to sarcoma care offers a visionary blueprint for the future, highlighting the synergy between AI and digital health in achieving a sustainable healthcare system. From a practical standpoint, this roadmap serves as a guide for healthcare providers, administrators and policymakers. It outlines actionable steps such as the adoption of interoperable digital platforms, the integration of AI in diagnostic and treatment protocols, and the establishment of quality indicators for continuous improvement. These practical measures aim to facilitate the transition from traditional, volume-based healthcare models to a more dynamic, value-based approach. The roadmap also suggests the use of real-world-time data to validate and refine AI algorithms, thereby ensuring that technological advancements are rooted in tangible clinical benefits. The roadmap outlines a multi-faceted approach that includes real-world-time data collection, interoperable digital platforms for data management, automated analysis employing AI algorithms, and benchmarking against quality indicators specific to sarcoma care [19,26]. These elements come together to assess various dimensions of care, including clinical outcomes and patient experiences. The ultimate aim is to continuously refine sarcoma care through iterative improvements, bringing the healthcare system closer to realizing value-based precision care. Adding another layer of innovation, the roadmap aims to incorporate the concept of a sarcoma digital twin, a virtual replica of an individual patient’s medical condition that integrates seamlessly with AI-driven predictive modeling. As the roadmap evolves, there will be an increasing focus on aligning costs with value, thereby contributing to a more sustainable, efficient, and patient-centric healthcare system. Thus, the roadmap represents not just a pathway for sarcoma care but also serves as a model for the broader application of precision medicine and value-based healthcare.

While the Swiss Sarcoma Network provides a innovative model, its potential is not confined to Switzerland alone. By fostering international collaborations and partnerships, this roadmap can be scaled globally, adapted to diverse healthcare infrastructures and socio-cultural contexts. Key to this expansion is the network’s emphasis on interoperability and standardization, facilitating seamless data exchange across borders. The establishment of international sarcoma care consortiums, working cohesively within the potential of such platform, can harmonize methodologies, share best practices, and collectively advance the vision of precision medicine. As more regions adopt this model, there is an opportunity for global real-world-time data aggregation, enhancing AI’s predictive capabilities and refining treatment strategies. Thus, the roadmap represents not just a pathway for sarcoma care but also serves as a model for the broader application of precision medicine and value-based healthcare on a global scale.

## 7. Ethical and Regulatory Forethought

As we advance toward a new paradigm of value-based precision care in sarcoma, underpinned by digital health and AI, ethical and regulatory considerations must be addressed with the same vigor as technological innovations [31,32]. The collection, storage, and analysis of patient data pose questions about data security, privacy, and informed consent. Ensuring equitable access to advanced sarcoma treatments catalyzed by AI and digital tools is paramount to preventing disparities in care. However, it is important to acknowledge the limitations of our approach. While AI and digital health platforms offer transformative potential, they are no without their drawbacks. The quality of AI algorithms is highly dependent on the quality and quantity of the data fed into them. Incomplete or biased data can lead to inaccurate or even harmful clinical decisions. Additionally, the ethical implications of AI decision making in healthcare are still not fully understood and require further study. There is also the risk of over-reliance on technology, which could potentially undermine the role of medical professionals in patient care. Furthermore, the cost of implementing advanced digital solutions may be prohibitive for smaller healthcare facilities, potentially widening the gap in the quality of care. Regulatory bodies and ethical committees must work in concert with healthcare providers, technology developers, and policymakers to standardize protocols, ensuring that they are universally applicable and ethically sound. These protocols must also be flexible enough to adapt to rapid technological advancements without compromising patient safety or data integrity. As real-world data platforms become more integrated into the healthcare system, legal frameworks will play a critical role in shaping the ethical landscape of digital health and AI in sarcoma care. Thus, ethical and regulatory forethought is not a mere afterthought but an integral component of the roadmap to value-based precision care.

## 8. Challenges and Barriers: A Call to Action

While the horizon is bright with the promise of digital health and AI ushering in a new era of value-based precision care in sarcoma, the path is fraught with challenges that require immediate attention. Practically speaking, the implementation of this roadmap will necessitate substantial investments in technology and human resources. Hospitals and healthcare providers will need to upgrade their existing infrastructures to support data-intensive AI algorithms. Training programs will be essential for clinicians to effectively interpret and act upon AI-generated insights. Moreover, the roadmap calls for a collaborative effort involving not just the medical community but also regulatory bodies and insurance providers. This multi-stakeholder approach is crucial for overcoming the financial, ethical, and logistical barriers to implementing a value-based healthcare model in sarcoma care. Resource constraints, a lack of standardized data protocols, and resistance to change within medical institutions all pose significant barriers. The dearth of expertise in data science within the medical community adds another layer of complexity. Furthermore, data privacy concerns and regulatory hurdles can slow down the pace of innovation. However, these challenges should not deter us; rather, they should serve as a clarion call to action. This involves not only healthcare professionals and technologists but also policymakers, patient advocacy groups, and regulatory bodies. A collective, multidisciplinary effort is crucial to overcome these barriers. Funding must be allocated for research and development, educational initiatives must be put in place, and policy frameworks need to be developed to encourage data sharing and interoperability. By acknowledging and addressing these challenges head-on, we can accelerate the journey toward realizing the full potential of digital health and AI in transforming sarcoma care.

## 9. Conclusions and Proposals for Future Research

In the evolving landscape of sarcoma care, the convergence of digital health and artificial intelligence offers a beacon of hope for personalized, efficient, and value-based treatment options. We have explored the promise this union holds—from the integration of real-world-time data and interoperable digital platforms to the application of AI for predictive analytics, all the way to the conceptualization of the sarcoma digital twin, thereby enabling predictive and value-based precision sarcoma care. As we look to the future, several avenues for research emerge. First, there is a need for randomized controlled trials (or, alternatively, target trial emulations) to validate the efficacy of AI algorithms in sarcoma diagnosis and treatment planning. Second, research should focus on the ethical implications of AI in healthcare, particularly in the context of data privacy and informed consent. Third, the economic aspects of implementing digital health platforms and AI in sarcoma care warrant in-depth study, including cost–benefit analyses and long-term sustainability assessments. Lastly, future work could explore the integration of other emerging technologies, such as blockchain for secure data sharing or augmented reality for enhanced surgical planning, into the existing digital health ecosystem. These research proposals aim to fill the existing gaps in our understanding and provide a comprehensive framework for the adoption of digital health and AI in sarcoma care. While the challenges are significant, they are not insurmountable. We stand at the cusp of a transformative era in healthcare, one where the systematic adoption of these technologies could revolutionize the way we approach not just sarcoma, but complex diseases at large. However, to realize this vision, a coordinated, multidisciplinary effort is essential. The time for action is now; let us seize this moment to propel sarcoma care into a future replete with the benefits of digital health and AI, ultimately improving outcomes and quality of life for patients around the globe.

## Data Availability

Not applicable.

## References

[1] Kubicek P., Cesne A.L., Lervat C., Toulmonde M., Chevreau C., Duffaud F., Le Nail L.-R., Morelle M., Gaspar N., Vérité C. (2023). Management and outcomes of adolescent and young adult sarcoma patients: Results from the French nationwide database NETSARC. BMC Cancer.

[2] Blay J.-Y., Penel N., Gouin F., Le Cesne A., Toulmonde M. (2022). Improving at a nationwide level the management of patients with sarcomas with an expert network. Ann. Oncol..

[3] Blay J.-Y., Casali P., Bouvier C., Dehais C., Galloway I., Gietema J., Halámková J., Hindi N., Idbaih A., Kinloch E. (2021). European Reference Network for rare adult solid cancers, statement and integration to health care systems of member states: A position paper of the ERN EURACAN. ESMO Open Cancer Horizons.

[4] Blay J.Y., Bonvalot S., Gouin F., Le Cesne A., Penel N. (2019). Criteria for reference centers for sarcomas: Volume but also long-term multidisciplinary organisation. Ann. Oncol..

[5] Blay J.Y., Soibinet P., Penel N., Bompas E., Duffaud F., Stoeckle E., Mir O., Adam J., Chevreau C., Bonvalot S. (2017). Improved survival using specialized multidisciplinary board in sarcoma patients. Ann. Oncol..

[6] Hoeben A., Joosten E.A.J., van den Beuken-van Everdingen M.H.J. (2021). Personalized Medicine: Recent Progress in Cancer Therapy. Cancers.

[7] Jameson J.L., Longo D.L. (2015). Precision medicine—Personalized, problematic, and promising. N. Engl. J. Med..

[8] Denny J.C., Collins F.S. (2021). Precision medicine in 2030-seven ways to transform healthcare. Cell.

[9] Porter M.E. (2009). A strategy for health care reform—Toward a value-based system. N. Engl. J. Med..

[10] Porter M.E., Pabo E.A., Lee T.H. (2013). Redesigning primary care: A strategic vision to improve value by organizing around patients’ needs. Health Aff..

[11] Obermeyer Z., Emanuel E.J. (2016). Predicting the Future—Big Data, Machine Learning, and Clinical Medicine. N. Engl. J. Med..

[12] Obermeyer Z., Topol E.J. (2021). Artificial intelligence, bias, and patients’ perspectives. Lancet.

[13] Chen J.H., Asch S.M. (2017). Machine Learning and Prediction in Medicine—Beyond the Peak of Inflated Expectations. N. Engl. J. Med..

[14] Zilchman E., Nicklin W., Aggarwal R., Bates D. (2021). Health Care 2030: The coming transformatrion. NEJM Catalyst.

[15] Fuchs B., Studer G., Bode B., Wellauer H., Frei A., Theus C., Schupfer G., Plock J., Windegger H., Breitenstein S. (2021). Development of a value-based healthcare delivery model for sarcoma patients. Swiss Med. Wkly..

[16] Kaplan R.S., Porter M.E. (2011). How to solve the cost crisis in health care. Harv. Bus. Rev..

[17] Blay J.Y., Hindi N., Bollard J., Aguiar S., Angel M., Araya B., Badilla R., Bernabeu D., Campos F., Caro-Sánchez C.H.S. (2022). SELNET clinical practice guidelines for soft tissue sarcoma and GIST. Cancer Treat. Rev..

[18] Blay J.Y., Palmerini E., Bollard J., Aguiar S., Angel M., Araya B., Badilla R., Bernabeu D., Campos F., Chs C.S. (2022). SELNET Clinical practice guidelines for bone sarcoma. Crit. Rev. Oncol. Hematol..

[19] Fuchs B., Schelling G., Elyes M., Studer G., Bode-Lesniewska B., Scaglioni M.F., Giovanoli P., Heesen P. (2023). Unlocking the Power of Benchmarking: Real-World-Time Data Analysis for Enhanced Sarcoma Patient Outcomes. Cancers.

[20] Bates D.W. (2023). How to regulate evolving AI health algorithms. Nat. Med..

[21] Haug C.J., Drazen J.M. (2023). Artificial Intelligence and Machine Learning in Clinical Medicine, 2023. N. Engl. J. Med..

[22] Heesen P., Studer G., Bode B., Windegger H., Staeheli B., Aliu P., Martin-Broto J., Gronchi A., Blay J.Y., Le Cesne A. (2022). Quality of Sarcoma Care: Longitudinal Real-Time Assessment and Evidence Analytics of Quality Indicators. Cancers.

[23] Topol E.J. (2019). High-performance medicine: The convergence of human and artificial intelligence. Nat. Med..

[24] Rajpurkar P., Chen E., Banerjee O., Topol E.J. (2022). AI in health and medicine. Nat. Med..

[25] Steinhubl S.R., Muse E.D., Topol E.J. (2013). Can mobile health technologies transform health care?. JAMA.

[26] Esteva A., Robicquet A., Ramsundar B., Kuleshov V., DePristo M., Chou K., Cui C., Corrado G., Thrun S., Dean J. (2019). A guide to deep learning in healthcare. Nat. Med..

[27] Jiang F., Jiang Y., Zhi H., Dong Y., Li H., Ma S., Wang Y., Dong Q., Shen H., Wang Y. (2017). Artificial intelligence in healthcare: Past, present and future. Stroke Vasc. Neurol..

[28] Bera K., Braman N., Gupta A., Velcheti V., Madabhushi A. (2021). Predicting cancer outcomes with radiomics and artificial intelligence in radiology. Nat. Rev. Clin. Oncol..

[29] Hernandez-Boussard T., Macklin P., Greenspan E.J., Gryshuk A.L., Stahlberg E., Syeda-Mahmood T., Shmulevich I. (2021). Digital twins for predictive oncology will be a paradigm shift for precision cancer care. Nat. Med..

[30] Mosku N., Heesen P., Christen S., Scaglioni M.F., Bode B., Studer G., Fuchs B. (2023). The Sarcoma-Specific Instrument to Longitudinally Assess Health-Related Outcomes of the Routine Care Cycle. Diagnostics.

[31] Char D.S., Shah N.H., Magnus D. (2018). Implementing Machine Learning in Health Care—Addressing Ethical Challenges. N. Engl. J. Med..

[32] Price W.N., Cohen I.G. (2019). Privacy in the age of medical big data. Nat. Med..

